# Supplementary material for: Large scale active-learning-guided exploration for in vitro protein production optimization
Source: Nat Commun. 2020 Apr 20;11:1872. doi: 10.1038/s41467-020-15798-5 (PMC7170859; doi:10.1038/s41467-020-15798-5)
Supplement: Supplementary file 1 — Supplementary Information [file 41467_2020_15798_MOESM1_ESM.pdf]

Supplementary Information

Title

Large scale active-learning-guided exploration for *in vitro* protein production optimization

Authors

Olivier Borkowski[1,5], Mathilde Koch[,2,5], Agnès Zettor[3], Amir Pandi[2], Angelo Cardoso Batista[2], Paul Soudier[2] and Jean-Loup Faulon[1,2,4*]

[1]Génomique Métabolique, Genoscope, Institut François Jacob, CEA, CNRS, Univ Evry, Université Paris-Saclay, 91057 Evry, France

[2]Micalis Institute, INRA, AgroParisTech, Université Paris-Saclay, Jouy-en-Josas, France

[3]Chemogenomic and Biological Screening Core Facility, Institut Pasteur, Department of Structural Biology and Chemistry, center for technological resources and research (C2RT) 25/28 rue du Dr Roux, 75724 Paris cedex 15, France.

[4]SYNBIOCHEM Center, School of Chemistry, University of Manchester, Manchester, UK

5These authors contributed equally: Olivier Borkowski, Mathilde Koch

*e-mail: jean-loup.faulon@inra.fr

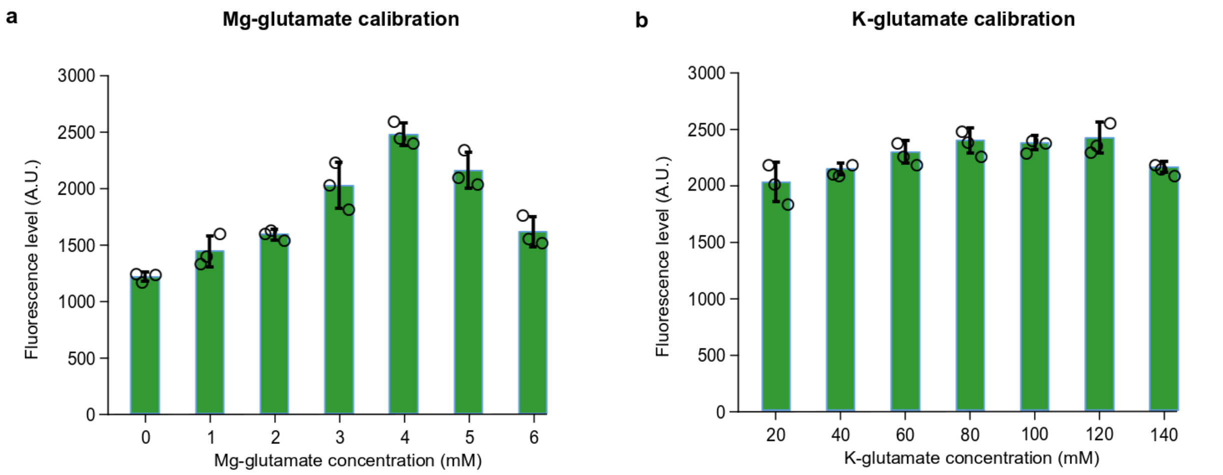

Supplementary Figure 1: Preliminary calibration of the cell-free composition. The lysate is usually only calibrated for Mg-glutamate, K-glutamate levels. Here we show the end point after overnight cell-free reactions with the lysate_ORI used in **Fig. 1**. Then, we fixed the maximum concentration for: **a,** Mg-glutamate concentration at 4 mM and **b,** K-glutamate at 80 mM. The error bars stand for the standard deviation of 3 replicates performed on the same day. Data are mean values and the vertical black lines stand for the standard deviation of the 3 replicates (n=3 independent experiments)

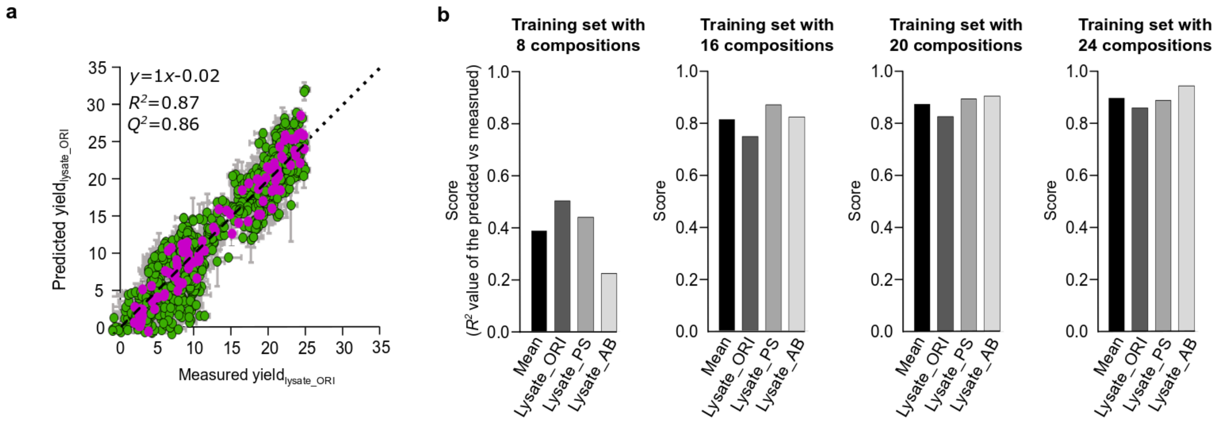

Supplementary Figure 2: The choice of 102 cell-free compositions for training and testing of our model. a, Distribution of the yields obtained with the 102 training cell-free compositions along the 1017 cell-free compositions tested in **fig 1**. The 102 cell-free compositions were chosen based on the highest R^2 obtained by training on 102 points and predicting on the 915 remaining points. The data are mean values and the vertical error bars stand for the standard deviation of 3 replicates. The horizontal error bars stand for the standard deviation of 25 predictions. **b**, Comparison of the prediction efficiency of the model when trained with a training set of 8, 16, 20 or 24 cell-free compositions, for prediction on the reminder of the 102 points. The training set is chosen amongst the 102 cell-free compositions fixed in **panel a**. The training set leading to the highest mean R^2 amongst the 3 lysates has been selected.

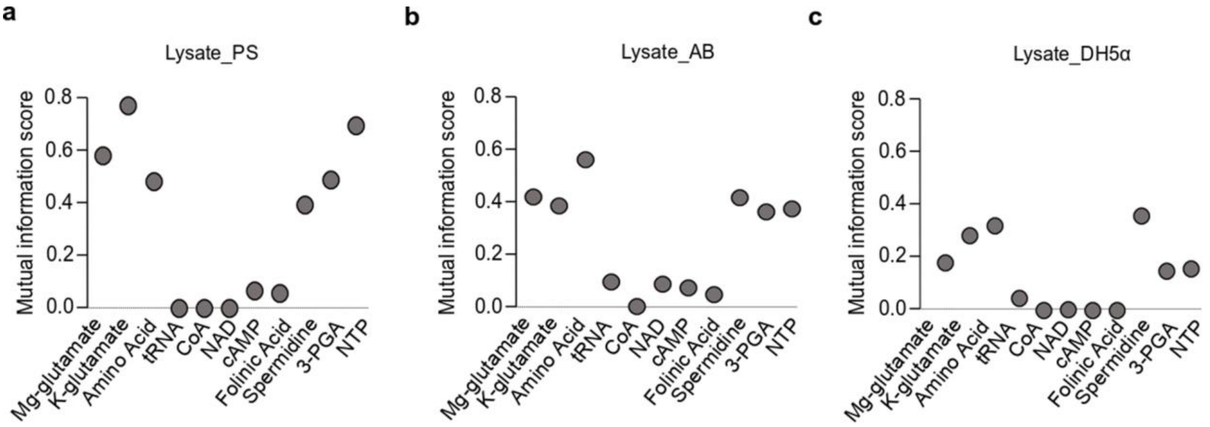

Supplementary Figure 3: Mutual Information analysis based on the 102 compositions tested with lysate-PS, lysate_AB and lysate_DH5α. Mutual information analysis of the relationship between the yield and each chemical compound, using the yields measured in cell-free reactions using 102 cell-free compositions and **a**, lysate_PS, **b**, lysate_AB, **c**, lysate_DH5α.

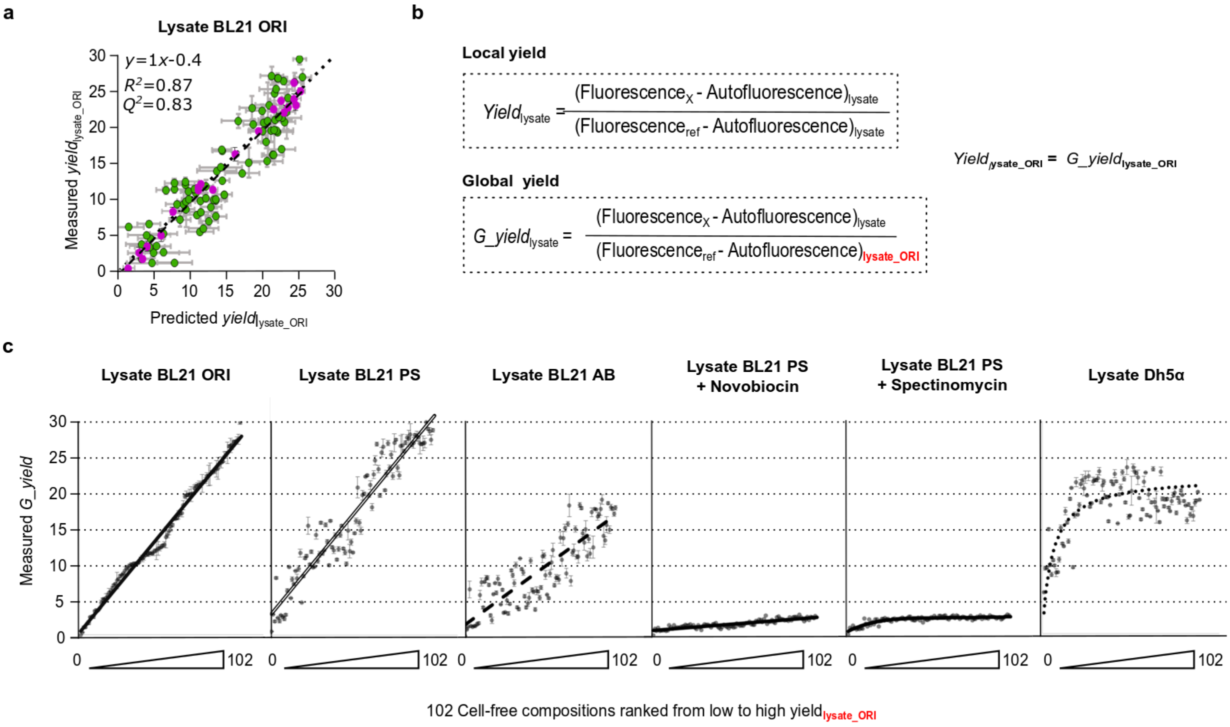

Supplementary Figure 4: Global comparison between the yields obtained with different lysates. a, Comparison of the yields obtained with the lysate original (same as **fig. 1**) vs the model predictions for the 102 cell-free compositions used in **Fig. 2**. The data are mean values and the horizontal error bars stand for the standard deviation of 3 replicates. The vertical error bars stand for the standard deviation of 25 predictions. **b**, Formula of the global yield compared to the local yield. In contrary to the Yields presented in Fig 2., the Global yield always use the same reference yield from the lysate of **Fig. 1** named Lysate_ORI. The Global yield, noted G_yield, allows comparison between yields obtained with our different lysates. **c**, The 102 cell-free compositions were ranked from low to high values based on the yields obtained with the Lysate_ORI. The same ranking of the same 102 cell-free compositions was used for each lysate. Linear fit is used for Lysate_ORI, Lysate_PS, Lysate_AB and Lysate_PS + novobiocin. Michaelis-Menten like fit is used for Lysate_PS + Spectinomycin and Lysate_DH5α.

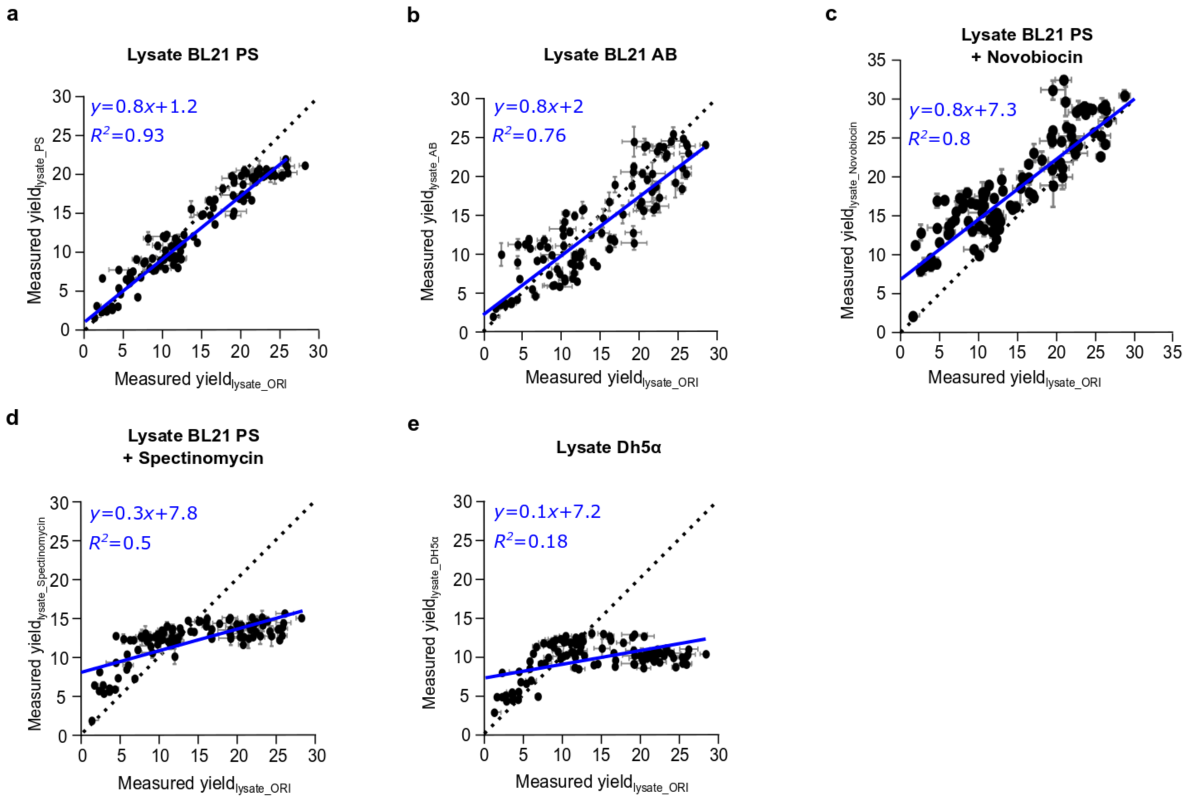

Supplementary Figure 5: Comparison between the behaviour of the local yields measured with different lysates and the yields measured with the lysate_ORI. Comparison between the yields measured with Lysate_ORI and **a,** Lysate_PS. **b,** Lysate_AB. **c,** Lysate_PS + novobiocin. **d,** Lysate_PS + spectinomycin. **e,** Lysate_DH5α. The blue lines stand for linear fit and the dot lines stand for the perfect correlation (intercept 0 and slope 1). We used the same 102 cell-free compositions for all the measurements. The error bars stand for the standard deviation of 3 replicates In all panels, the data are mean values and the horizontal and vertical error bars stand for the standard deviation of 3 replicates.

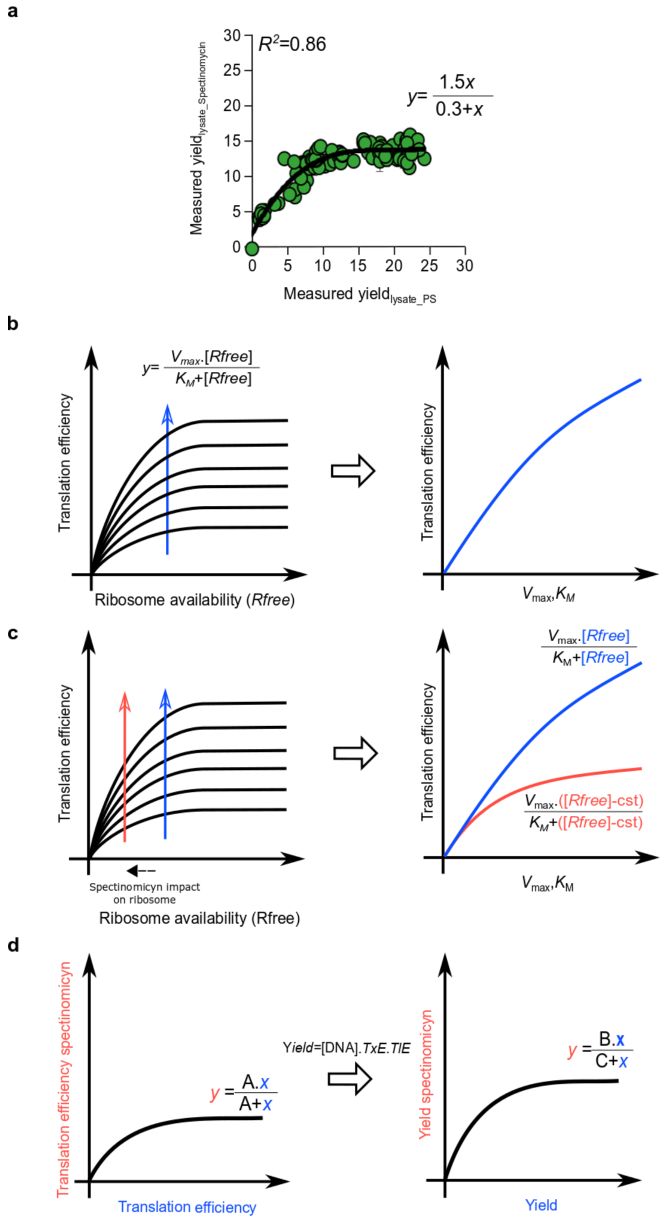

Supplementary Figure 6: A decrease in ribosome availability is sufficient to explain the saturation of the yields with Lysate_Spectinomycin . a, Comparison between the yield obtained with Lysate_PS and the yield obtained with Lysate_PS supplemented with Spectinomycin (same data as **Supplementary Fig. 5d**). We used a Michaelis-Menten like function to fit the data. **b**, We used the well described Michaelis-Menten[1] like relationship between translation efficiency and available ribosomes concentration (Rfree). We assumed that a change in cell-composition impact the translation efficiency via a change of V_{max} and K_M. At a fixed *Rfree* concentration (blue arrow), an increase of V_{max},K_M values lead to an increasing translation efficiency. **c**, As the spectinomycin binds to the 30S subunit of the ribosome to inhibit the translation process, its activity can be represented by a decrease in *Rfree* concentration (red arrow). The impact of less ribosomes will lead to a decrease in translation efficiency (blue vs red line in the second plot). **d**, Relationship between a translation efficiency with spectinomycin versus a translation efficiency without spectinomycin (see **supplementary note 2**). The yield as the protein production results from the translation but also the transcription process. The relationship between Translation efficiency and yields is described in **supplementary note 2.**

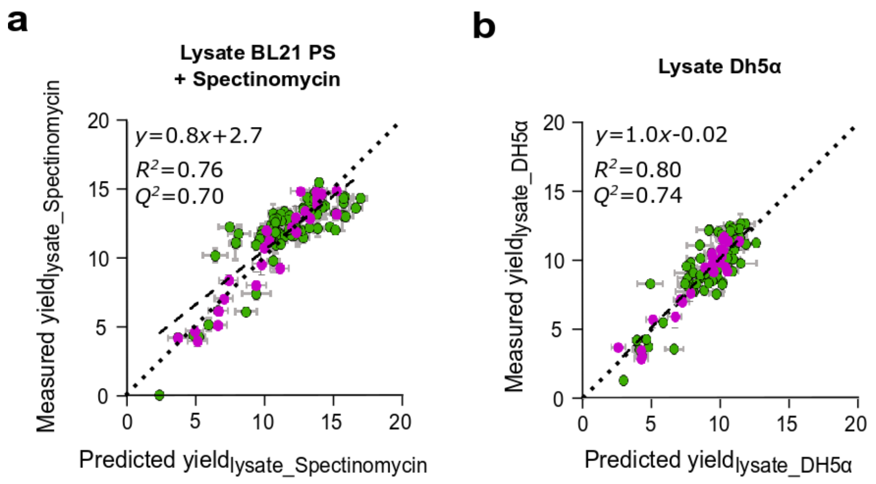

Supplementary Figure 7: Predictions using a training set of 25 buffer compositions. Both training sets are using the same 25 buffer compositions. **a,** Comparison of the yields obtained with the lysate BL21 PS**,** supplemented with 0.5 mg.ml^{-1} of spectinomycin vs. the model predictions. **b,** Comparison of the yields obtained with a lysate obtained from the stain DH5a vs. the model predictions. The model predictions are based on the model used in Figure 4 trained with 20 buffer compositions to which 5 composition were added. The 5 compositions added to the training set were selected following an exploration strategy and correspond to the compositions for which the model exhibited the highest standard deviation. In all panels, data are mean values and the horizontal grey lines stand for the standard deviation of 3 replicates. The vertical grey lines stand for the standard deviation of 25 predictions. R^2 value was computed on 102 values, Q^2 on the 77 values of the test set, the linear regression fits the 77 test values

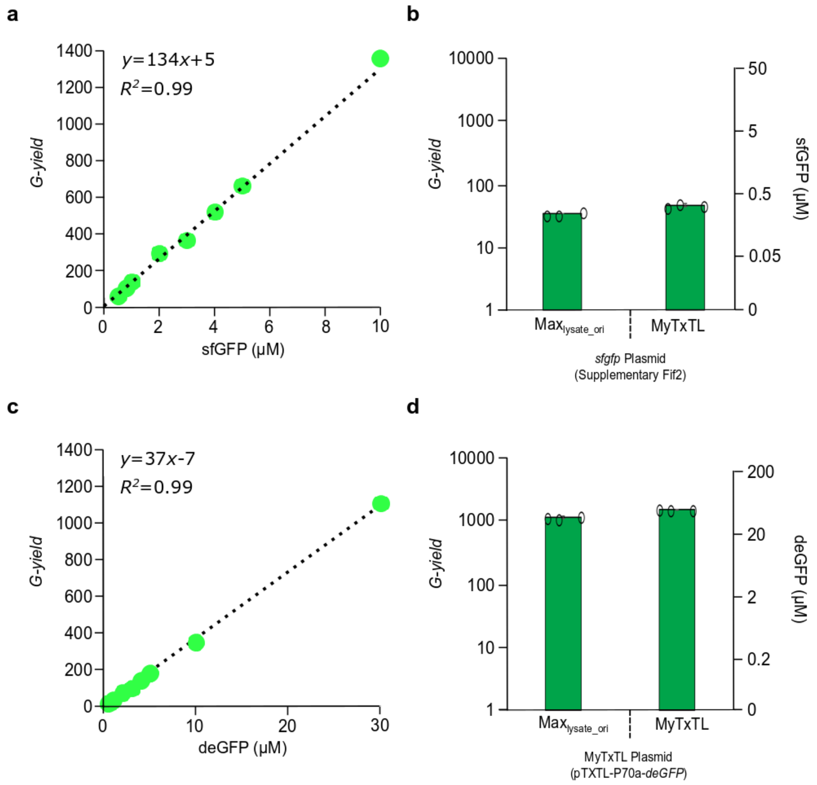

Supplementary Figure 8: Absolute measurements in cell-free reaction
a, Relationship between purified sfGFP and the Global yield. (See **supplementary note 1**). **b**, Comparison between the yield obtained with our best cell-free composition with lysate_ORI and the commercial kit myTXTL from Arbor using our plasmid. Data are mean values and the vertical black lines stand for the standard deviation of the 3 replicates (n=3 independent experiments). **c**, Relationship between purified deGFP and the Global yield. **d**. Comparison between the yield obtained with our best cell-free composition with lysate_ORI and the commercial kit myTXTL from Arbor using myTXTL plasmid (pTXTL-P70a(2)-*deGFP*). Data are mean values and the vertical black lines stand for the standard deviation of the 3 replicates (n=3 independent experiments)

Supplementary Table 1: Sequence of the plasmid used in this study.

Promoter J23101	tttacagctagctcagtcctaggtattatgctagc
RBS B0034	aaagaggagaaa
sfgp	atgcgtaaaggcgaagagctgttcactggtgtcgtccctattctggtgga<br>actggatggtgatgtcaacggtcataagttttccgtgcgtggcgagggtg<br>aaggtgacgcaactaatggtaaactgacgctgaagttcatctgtactact<br>ggtaaactgccggtaccttggccgactctggtaacgacgctgacttatgg<br>tgttcagtgctttgctcgttatccggaccatatgaagcagcatgacttct<br>tcaagtccgccatgccggaaggctatgtgcaggaacgcacgatttccttt<br>aaggatgacggcacgtacaaaacgcgtgcggaagtgaaatttgaaggcga<br>taccctggtaaaccgcattgagctgaaaggcattgactttaaagaagacg<br>gcaatatcctgggccataagctggaatacaattttaacagccacaatgtt<br>tacatcaccgccgataaacaaaaaaatggcattaaagcgaattttaaaat<br>tcgccacaacgtggaggatggcagcgtgcagctggctgatcactaccagc<br>aaaacactccaatcggtgatggtcctgttctgctgccagacaatcactat<br>ctgagcacgcaaagcgttctgtctaaagatccgaacgagaaacgcgatca<br>tatggttctgctggagttcgtaaccgcagcgggcatcacgcatggtatgg<br>atgaactgtacaaatga
rrnB T1 terminator	ccaggcatcaaataaaacgaaaggctcagtcgaaagactgggcctttcgt<br>tttatctgttgtttgtcggtgaacgctctc

The *sfgp* is under control of the promoter J23101 (http://parts.igem.org/Part:BBa_J23101) and RBS B0034 (http://parts.igem.org/Part:BBa_B0034). The plasmid contains the gene of ampicillin resistance and the origin of replication PBR322.

Supplementary Table 2: Buffer compositions of the Training sets.

	Mg-glutamate (mM)	K-glutamate (mM)	Amino Acid (mM)	tRNA (mg.ml^{-1})	CoA (mM)	NAD (mM)	cAMP (mM)	Folinic Acid (mM)	Spermidine (mM)	3-PGA (mM)	NTP (mM)
**20 initial buffer Compositions**	4	80	0.45	0.1	0.026	0.033	0.075	0.007	1	9	1.5
	4	80	0.45	0.06	0.078	0.033	0.075	0.007	1	9	1.5
	4	80	1.5	0.2	0.26	0.33	0.75	0.068	0.1	30	1.5
	1.2	8	0.15	0.02	0.026	0.033	0.075	0.02	0.3	3	0.75
	1.2	40	0.15	0.06	0.13	0.099	0.075	0.034	0.3	15	0.75
	0.4	24	0.45	0.1	0.078	0.165	0.225	0.02	0.3	9	0.45
	2	40	0.15	0.1	0.026	0.033	0.225	0.007	0.3	15	0.45
	2	40	0.45	0.1	0.026	0.033	0.225	0.034	0.3	15	0.75
	2	40	0.45	0.02	0.026	0.099	0.225	0.034	0.3	15	0.75
	2	40	0.45	0.1	0.078	0.033	0.075	0.034	0.1	9	0.75
	4	80	0.15	0.2	0.026	0.099	0.075	0.007	0.3	9	1.5
	4	80	0.15	0.02	0.13	0.033	0.075	0.034	0.3	9	1.5
	2	80	1.5	0.1	0.026	0.033	0.075	0.02	0.1	9	1.5
	4	80	0.45	0.02	0.078	0.165	0.075	0.007	0.1	9	1.5
	4	80	1.5	0.1	0.078	0.165	0.375	0.034	0.1	9	1.5
	4	80	0.75	0.2	0.078	0.033	0.075	0.007	0.1	9	1.5
	4	80	1.5	0.2	0.078	0.033	0.375	0.007	0.1	9	1.5
	4	80	1.5	0.02	0.026	0.033	0.225	0.02	0.1	9	1.5
	4	80	1.5	0.1	0.026	0.165	0.075	0.034	0.1	9	1.5
	4	80	1.5	0.06	0.026	0.099	0.075	0.02	0.1	9	1.5
**5 Extra buffer compositions**	2	8	0.75	0.06	0.026	0.099	0.375	0.007	0.1	15	0.15
	4	80	0.15	0.2	0.078	0.33	0.075	0.02	1	9	1.5
	0.4	40	0.75	0.06	0.026	0.033	0.075	0.02	0.5	3	0.75
	2	8	0.15	0.1	0.026	0.165	0.225	0.034	0.5	9	0.45
	1.2	40	0.45	0.02	0.026	0.033	0.075	0.02	0.3	3	0.75

SUPPLEMENTARY NOTE 1: Commercial kit and absolute sfGFP/deGFP measurements (Supplementary Figure 8)

Both plasmids (our plasmid and myTXL plasmid) led to similar yield when the lysate_ORI with the optimized composition (max yield in **Fig. 1d**) and myTXTL mix are used. This result suggests that pTXTL-P70a(2)-deGFP can also be used, instead of our plasmid to optimize cell-free composition. The higher Global yield come from the higher fluorescence obtained with this plasmid. The pTXTL-P70a(2)-deGFP seems to be a derivative of the pBEST-OR2-OR1-Pr-UTR1-eGFP-Del6-229-T500[1] optimize for expression in cell-free reaction. We don't have access to the cell-free composition of myTXTL mix but we assumed that it was optimized to obtain a maximum protein production and that the lysate was prepared from a modified strain of *E coli*. The quality of the result obtained with our lysate-specific optimization compared to the commercial kit is a validation of our method efficiency. The protein concentration obtained from the expression of our plasmid with lysate_ORI is at 0.22 µM sfGFP equivalent. Eventually with myTXTL plasmid from arbor, we obtained a deGFP concentration of 31 µM (~0.85 $mg.ml^{-1}$) and 42 µM (~1.2 $mg.ml^{-1}$) with our optimized cell-free composition and with MyTXTL cell-free reaction.

SUPPLEMENTARY NOTE 2: Deterministic model of protein production behavior in cell-free system with an imparied translation process (Supplementary Figure 6)

Assumption 1: Adding spectinomycin lead to a similar impact on the translation process as a decrease in concentration of the available ribosome. Spectinomycin binds to the 30S subunit stopping protein synthesis. Thus, a subset of ribosomes should be unavailable for translation.
$[Rfree]_{spec}=[Rfree]$-cst.
Assumption 2: We simplified our calculation by considering that a variation in cell-free composition has a similar impact on both V_{max} and K_M.
V_{max} = cst_2 x K_M.
Assumption 3: The relationship of transcription efficiencies (noted *TxE*) between lysates is modelled by a linear relationship with a negligible intercept. We observed such a linear relationship (with an intercept close to 0) between yields from lysates with and without an impaired transcription machinery in **Supplementary Figure 5c.**
$TxE_{spec}=cst_3.TxE$
Assumption 4: The variation in cell-free composition mainly affects the translation process. We observed in **Supplementary Figure 5d** that a lysate with a damaged translation machinery is poorly improved by a change in cell-free composition. The opposite is observed with an inefficient transcription machinery in **Supplementary Figure 5c** suggesting that the efficiency of the translation machinery is the limiting factor for cell-free improvement and not the efficiency of the transcription machinery.
$TxE=cst_4$ (*TxE* is independent of the variations in cell-free compositions)

We used the well-defined model of the translation efficiency (*TlE*) based on a Michaelis-Menten equation[2]:

$$TlE = \frac{V_{max}.[Rfree]}{K_m+[Rfree]} \quad (1)$$

$$TlE_{spec} = \frac{V_{max}.[Rfree]_{spec}}{K_m+[Rfree]_{spec}} \quad (2)$$

where V_{max} and K_M values depends on the RBS sequence and the cell-free composition. [*Rfree*] stands for the concentration in available ribosomes.

Assumption 1: $[Rfree]_{spec}=[Rfree]$-cst.

$$(2) \Leftrightarrow TlE_{spec} = \frac{V_{max}.([Rfree]-cst)}{K_m+([Rfree]-cst)} \quad (3)$$

Assumption 2: V_{max} = cst_2 x K_M

$$(1) \Leftrightarrow TlE = \frac{cst_2.K_m.[Rfree]}{K_m+[Rfree]} \quad (4)$$

$$(3) \Leftrightarrow TlE_{spec} = \frac{cst_2.K_m.([Rfree]-cst)}{K_m+([Rfree]-cst)} \quad (5)$$

Thus,

$$(4) \Leftrightarrow K_M = \frac{TlE.[Rfree]}{cst_2.[Rfree]-TlE} \quad (6)$$

$$(5)\&(6) \Leftrightarrow TlE_{spec} = \frac{cst_2.\frac{TlE.[Rfree]}{cst_2.[Rfree]-TlE}.([Rfree]-cst)}{\frac{TlE.[Rfree]}{cst_2.[Rfree]-TlE}+([Rfree]-cst)}$$

$$\Leftrightarrow TlE_{spec} = \frac{cst_2.TlE.[Rfree].([Rfree]-cst)}{TlE.[Rfree]+([Rfree]-cst)(cst_2.[Rfree]-TlE)}$$

$$\Leftrightarrow TlE_{spec} = \frac{cst_2.TlE.[Rfree].([Rfree]-cst)}{TlE.cst+([Rfree]-cst)cst_2.[Rfree]}$$

$$\Leftrightarrow TlE_{spec} = \frac{\frac{cst_2.[Rfree].([Rfree]-cst)}{cst}.TlE}{TlE+\frac{cst_2.[Rfree].([Rfree]-cst)}{cst}}$$

$$\Leftrightarrow TlE_{spec} = \frac{A.TlE}{TlE+A} \text{ with } A = \frac{cst_2.[Rfree].([Rfree]-cst)}{cst} \quad (7)$$

The protein production (and so the yield) is the result of the expression of *sfgfp* by the transcription and translation processes.

$$Yield = [DNA].TxE.TlE \quad (8)$$
$$Yield_{spec} = [DNA].TxE_{spec}.TlE_{spec} \quad (9)$$

Assumption 3: $TxE_{spec}=cst_3.TxE$. Moreover, the DNA concentration is the same in every cell-free reaction so $[DNA]=cst_5$.

$$(9) \Leftrightarrow Yield_{spec} = cst_5.cst_3.TxE.TlE_{spec} \quad (10)$$

Assumption 4: $TxE=cst_4$

$$(8) \Leftrightarrow Yield = cst_5.cst_4.TlE \quad (11)$$
$$(10) \Leftrightarrow Yield_{spec} = cst_5.cst_3.cst_4.TlE_{spec} \quad (12)$$

Then,

$$(11) \Leftrightarrow \frac{Yield}{cst_5.cst_4} = TlE \quad (13)$$

and

$$(12) \Leftrightarrow \frac{Yield_{spec}}{cst_5.cst_3.cst_4} = TlE_{spec} \quad (14)$$

Then,

$$(14\&7) \Leftrightarrow \frac{Yield_{spec}}{cst_5.cst_3.cst_4} = \frac{A.TlE}{TlE+A} \quad (15)$$

Then,

$$(15\&11) \Leftrightarrow \frac{Yield_{spec}}{cst_5.cst_3.cst_4} = \frac{A.\frac{Yield}{cst_5.cst_4}}{\frac{Yield}{cst_5.cst_4}+A}$$

$$\Leftrightarrow Yield_{spec} = \frac{cst_5.cst_3.cst_4.A.\frac{Yield}{cst_5.cst_4}}{\frac{Yield}{cst_5.cst_4}+A}$$

$$\Leftrightarrow Yield_{spec} = \frac{cst_5.\, cst_3.\, cst_4.\, A.\, Yield}{Yield + A.\, cst_5.\, cst_4}$$

$$\Leftrightarrow Yield_{spec} = \frac{B.Yield}{Yield+C} \quad (16)$$

With $B = \frac{cst_5.cst_3.cst_4.cst_2.[Rfree].([Rfree]-cst)}{cst}$ and $C = \frac{cst_5.cst_4.cst_2.[Rfree].([Rfree]-cst)}{cst}$

Eventually, we obtained a Michaelis-Menten equation for the relationship between Yield and $Yield_{spec}$ (eq. 16) which explain the data in **Supplementary Fig. 6a**. Despite the multiple assumptions (that are difficult to verify by experimental measurements) this model gives a simple explanation of our observations.

References

1. Shin J., & Noireaux V. Efficient cell-free expression with the endogenous E. Coli RNA polymerase and sigma factor 70. *Journal Biological Engineering* **4**, 8 (2010).
2. Koch, M., Faulon J-L. & Borkowski O. Models for cell-free synthetic biology: make prototyping easier, better and faster. *Front Bioeng Biotechnol*. **6**, 182 (2018).
