# Supplementary material for: Large scale active-learning-guided exploration for in vitro protein production optimization
Source: Nat Commun. 2020 Apr 20;11:1872. doi: 10.1038/s41467-020-15798-5 (PMC7170859; doi:10.1038/s41467-020-15798-5)
Supplement: Supplementary file 3 — Reporting Summary [file 41467_2020_15798_MOESM3_ESM.pdf]

nature research

Corresponding author(s): Jean-Loup Faulon

Last updated by author(s): Mar 23, 2020

Reporting Summary

Nature Research wishes to improve the reproducibility of the work that we publish. This form provides structure for consistency and transparency in reporting. For further information on Nature Research policies, see Authors & Referees and the Editorial Policy Checklist .

Statistics

For all statistical analyses, confirm that the following items are present in the figure legend, table legend, main text, or Methods section.

n/a	Confirmed	
☐	☒	The exact sample size (*n*) for each experimental group/condition, given as a discrete number and unit of measurement
☐	☒	A statement on whether measurements were taken from distinct samples or whether the same sample was measured repeatedly
☐	☒	The statistical test(s) used AND whether they are one- or two-sided<br>*Only common tests should be described solely by name; describe more complex techniques in the Methods section.*
☒	☐	A description of all covariates tested
☒	☐	A description of any assumptions or corrections, such as tests of normality and adjustment for multiple comparisons
☐	☒	A full description of the statistical parameters including central tendency (e.g. means) or other basic estimates (e.g. regression coefficient) AND variation (e.g. standard deviation) or associated estimates of uncertainty (e.g. confidence intervals)
☒	☐	For null hypothesis testing, the test statistic (e.g. *F*, *t*, *r*) with confidence intervals, effect sizes, degrees of freedom and *P* value noted<br>*Give P values as exact values whenever suitable.*
☒	☐	For Bayesian analysis, information on the choice of priors and Markov chain Monte Carlo settings
☒	☐	For hierarchical and complex designs, identification of the appropriate level for tests and full reporting of outcomes
☒	☐	Estimates of effect sizes (e.g. Cohen's *d*, Pearson's *r*), indicating how they were calculated

Our web collection on statistics for biologists contains articles on many of the points above.

Software and code

Policy information about availability of computer code

Data collection	All scripts were written in Python (version 3.6.5), executed in Jupyter notebooks (version 1.0.0). Scripts are available online at github (https://github.com/brsynth/active_learning_cell_free). The libraries numpy and csv were used to handle files between different scripts. We used scitkit-learn22 (version 0.19.1) for all model training. An excel file (suppl data 1) is provided with all data used in the figures.
Data analysis	All scripts were written in Python (version 3.6.5), executed in Jupyter notebooks (version 1.0.0). Scripts are available online at github (https://github.com/brsynth/active_learning_cell_free). The libraries numpy and csv were used to handle files between different scripts. We used scitkit-learn22 (version 0.19.1) for all model training. Mean and STD were calculated with Excel.

For manuscripts utilizing custom algorithms or software that are central to the research but not yet described in published literature, software must be made available to editors/reviewers. We strongly encourage code deposition in a community repository (e.g. GitHub). See the Nature Research guidelines for submitting code & software for further information.

Data

Policy information about availability of data

All manuscripts must include a data availability statement. This statement should provide the following information, where applicable:

- Accession codes, unique identifiers, or web links for publicly available datasets
- A list of figures that have associated raw data
- A description of any restrictions on data availability

Data generated during the study are provided with the manuscript in an excel file (suppl data 1). A data availbility section can be find in the manuscript.

Field-specific reporting

Please select the one below that is the best fit for your research. If you are not sure, read the appropriate sections before making your selection.

- [x] Life sciences
- [ ] Behavioural & social sciences
- [ ] Ecological, evolutionary & environmental sciences

For a reference copy of the document with all sections, see nature.com/documents/nr-reporting-summary-flat.pdf

Life sciences study design

All studies must disclose on these points even when the disclosure is negative.

Sample size	The sample size was determined by the machine learning training. 711 points were used to obtain accurate predictions (regression coefficient > 0.9). 306 extra points were measured to check the prediction accuracy. In addition we measured 102 points with each extra lysate.
Data exclusions	We performed outlier removal based on the following criteria: if the coefficient of variation, amongst 3 replicates, was higher than 30%. This concerned 27 values of our 1017. This is apparent in the files as the value of the removed points appears as -1
Replication	Every measure was done in triplicate.
Randomization	For each lysate we used cell-free one organism. The randomization does not apply to this study.
Blinding	Instructions were provided by the algorithm and chemicals were distributed by a liquid handler independently of the experimentalist.

Reporting for specific materials, systems and methods

We require information from authors about some types of materials, experimental systems and methods used in many studies. Here, indicate whether each material, system or method listed is relevant to your study. If you are not sure if a list item applies to your research, read the appropriate section before selecting a response.

Materials & experimental systems

n/a	Involved in the study	
[x]	[ ]	Antibodies
[x]	[ ]	Eukaryotic cell lines
[x]	[ ]	Palaeontology
[x]	[ ]	Animals and other organisms
[x]	[ ]	Human research participants
[x]	[ ]	Clinical data

Methods

n/a	Involved in the study	
[x]	[ ]	ChIP-seq
[x]	[ ]	Flow cytometry
[x]	[ ]	MRI-based neuroimaging
