# Supplementary material for: Large scale active-learning-guided exploration for in vitro protein production optimization
Source: Nat Commun. 2020 Apr 20;11:1872. doi: 10.1038/s41467-020-15798-5 (PMC7170859; doi:10.1038/s41467-020-15798-5)
Supplement: Supplementary file 4 — Description of Additional Supplementary Files [file 41467_2020_15798_MOESM4_ESM.pdf]

Title: Supplementary Data 1

Description: Experimental data used to generate the figures in this work
